# Identifying HEXACO personality types: what do type characteristics tell us about student misconduct?

**DOI:** 10.1186/s12909-025-07599-5

**Published:** 2025-07-19

**Authors:** Ana Cristina Veríssimo, Pedro Oliveira, Paula Mena Matos, Laura Ribeiro

**Affiliations:** 1 Department of Public Health and Forensic Sciences and Medical Education, Medical Education Unit, Faculty of Medicine, University of Porto, Al. Prof. Hernâni Monteiro, Porto, 4200-319 Portugal; 2 ICBAS – School of Medicine and Biomedical Sciences, University of Porto, Porto, Portugal; 3 EPIUnit – Instituto de Saúde Pública, Universidade do Porto, Porto, Portugal; 4 Faculty of Psychology and Education Sciences, University of Porto, Porto, Portugal; 5 Center for Psychology at University of Porto, Porto, Portugal; 6 I3S – Instituto de Investigação e Inovação em Saúde, Universidade do Porto, Porto, Portugal

**Keywords:** Academic integrity, Higher education students, HEXACO personality types, Cluster analysis

## Abstract

**Background:**

While previous research has largely focused on individual trait associations with academic misbehaviour, this study used a person-centred approach to explore personality type differences in student misconduct. The distribution of the personality types was also compared across sociodemographic groups and fields of study.

**Methods:**

A sample of 1,398 health and non-health university students replied to a multiple-choice questionnaire covering HEXACO personality traits, eight forms of academic misconduct, and background information. Personality types were explored through cluster analysis, and differences in their behaviour and characteristics were examined using chi-square test and ANOVA.

**Results:**

The five personality types identified differed in their self-reported academic misconduct. Risk-takers disclosed the highest levels of misconduct, followed by Performance-oriented students, who had competitive dispositions. Secure and Socially Considerate students exhibited more well-adjusted and prosocial traits, respectively, disclosing the lowest rates of misconduct. Insecure students scored higher than these two types on Plagiarism, combining traits associated with psychological distress that may affect their performance. The personality types also revealed gender and socioeconomic differences, and a more uniform distribution in health than in non-health fields.

**Conclusion:**

This study expands on past research by analysing a distinct set of personality types in association with academic misconduct. Understanding the psychosocial characteristics and field of study distribution of these types helped to propose tailored educational strategies to foster their compliance with academic integrity.

**Supplementary Information:**

The online version contains supplementary material available at 10.1186/s12909-025-07599-5.

## Background

Academic integrity is a cornerstone of education and is built upon values such as ethics, transparency, trust, fairness, respect and responsibility [1]. Despite institutional endeavours to uphold academic integrity, both long-standing [2–4] and recent [5–7] reports indicate that academic misconduct remains a prevalent concern requiring attention.

Academic misconduct, encompassing unethical behaviours such as exam cheating, fraud in academic work, and plagiarism [8–10], is becoming more sophisticated with the rise of digital tools that can be misused for cheating [11, 12]. Academic misconduct undermines the educational, scientific and social mission of the university, damaging its reputation. Students who resort to misconduct can gain an unfair advantage and fail to develop essential professional skills [3, 13]. The absence of detection and repercussions for academic misbehaviour also fosters lenient attitudes and normalises cheating [14, 15]. The result is an increased risk of misconduct extending into postgraduation and scientific and professional careers, where societal consequences are even more concerning [16–18].

Personality traits reflect cognitive, emotional and behavioural tendencies [19] that have been shown to influence student propensity for academic misconduct [15, 20, 21]. The six-factor model (HEXACO), particularly, offers a comprehensive representation of personality, largely encompassing the Five Factor Model (FFM) dimensions [22]. Additionally, its Honesty–Humility dimension inversely reflects most of the core element of the Dark Triad [22, 23].

Selfish and antisocial tendencies, along with excessive competitiveness and ambition, have been associated with low moral inhibition and a higher inclination to misbehave academically [15, 20, 24]. Conversely, students with curious, creative, and open-minded dispositions tend to be more learning-oriented than motivated towards achievement [15, 25]. These students also tend to perform better academically, potentially reducing their propensity for misconduct [19, 26]. Similarly, conscientious students are more task-oriented, organised, dutiful and self-disciplined. These dispositions often translate into a greater sense of moral obligation [19] and better academic performance [27], reducing the risk of cheating. Conversely, low self-control can increase academic procrastination and lead to cheating [28, 29]. Risk-seeking and impulsivity [30, 31], as well as vulnerability to performance-impairing anxiety [32, 33], can also drive academic misconduct.

Personality influences misconduct by playing a role in student motivations, self-efficacy, academic performance, and beliefs about the morality of cheating. Conventional research largely relies on variable-centred approaches, examining associations between individual personality traits and academic misconduct. However, person-centred approaches provide a complementary perspective by recognising the existence of homogeneous subgroups within a population that share similar personality characteristics (personality types) [34, 35]. Person-centred research has been employed in fields such as the social sciences, public health and medicine [36]. This approach offers a more nuanced understanding of personality complexities by considering the interplay of different trait combinations within personality types. Consequently, personality types are particularly valuable for studying domains potentially influenced by multiple traits [34], such as academic misconduct.

Previous studies have explored personality types of the HEXACO traits [35, 37], including in higher education students [38]. Globally, their types ranged from self-centred to considerate, confident to insecure, and well-adjusted to maladjusted, primarily reflecting psychological differences. At this level, comparing the distribution of personality types across sociodemographic groups and fields of study can provide additional insights into their characteristics (‘who they are’) and academic distribution (‘where they are’) [38]. For instance, consistent findings report associations of personality with gender [39] and field of study [40, 41].

Overall, despite the advantages of person-centred approaches to understanding the intricate contributions of personality to different outcomes [34, 38], very few studies [42, 43] have explored personality types in association with academic misconduct in university students. Therefore, this study aimed to answer the following research questions: (1) How many distinct personality types emerge from the HEXACO traits in a diverse sample of higher education students?, (2) What are the psychological characteristics of these types?, (3) How prevalent are the personality types in different sociodemographic groups and fields of study?, and (4) Do the personality types differ in self-reported academic misconduct? Attending to the relatively exploratory nature of this study, the authors are cautious in formulating hypotheses. However, based on previous findings, it is anticipated that: (1) the number and (2) characteristics of the HEXACO personality types in our sample will largely replicate previous research findings [37, 38]; (3) the distribution of the personality types will vary between different fields of study [40, 41] and, at least, some sociodemographic groups [37, 43], as well as (4) according to self-reported academic misconduct [29, 43].

## Methods

### Participants

The population of this cross-sectional study were undergraduate and integrated master’s students at the University of Porto, the second-largest university in the country. They were invited to participate in the study via a university-wide email sent by the rectory, which included the link to the online questionnaire. Of the 20,997 eligible students, 1,398 participated in this study. The majority were females (71.7%), with a mean age of 20.36 (SD = 3.72) years, and were enrolled in diverse academic years and fields of study. The participants benefitted from the opportunity to win one of 10 vouchers through a draw. They were assured of the confidentiality of the study, and their informed consent was obtained.

### Instruments

*HEXACO-60* [44] was implemented using the validated Portuguese version [45] to assess six dimensions of personality: Honesty-Humility (e.g., “I would never accept a bribe, even if it were very large”), Emotionality (e.g., “I feel like crying when I see other people crying”), Extraversion (e.g., “In social situations, I’m usually the one who makes the first move”), Agreeableness (e.g., “I rarely hold a grudge, even against people who have badly wronged me”), Conscientiousness (e.g., “I plan ahead and organise things to avoid scrambling at the last minute”), and Openness to Experience (subsequently referred to as Openness, e.g., “I like people who have unconventional views”). Each dimension was assessed using 10 items, positively and negatively formulated, rated from 1– “strongly disagree” to 5– “strongly agree”.

*Academic Misconduct Questionnaire (AMQ)* [46] was applied to assess eight forms of academic misconduct: (i) Plagiarism, (ii) Exam Cheating using notes/device, (iii) Exam Cheating with colleagues, (iv) Obtaining/providing information prior exams, and (v) Signature Forgery in attendance sheets assessed more common forms of misconduct; (vi) Fraud in Academic Work (e.g., data fabrication/falsification) and (vii) Impersonation (assessment) covered more serious misbehaviour, and the last dimension evaluated propensity to (viii) Not Reporting peer misconduct (peers impaired by alcohol/drugs). Each dimension was assessed using two to five items, which describe whether participants have 1– “never” or 2– “at least once” committed the misbehaviour during university. Additionally, three items covered student perceptions regarding the frequency of peer fraud, the severity of the institutional penalty for cheating, and their knowledge of the academic code of conduct, respectively. The items were rated on a scale ranging from 1– “never/none” to 5– “very often/severe/very high”.

Information was also collected on demographics (gender, age) and socioeconomic background, including the highest level of parental education (1– “elementary school or lower” to 4– “master’s/doctorate”), school funding concern (“My concern about my ability to support the costs to complete my degree is”: 1– “none” or 2– “some/high”), and type of secondary school attended (1– “public” or 2– “private”). Given the potentially sensitive nature of questions regarding gender, parental education and funding concern, participants were given the option to “choose not to answer”. Students also indicated the course and academic year they were attending.

### Statistical analysis

Descriptive statistics (means and frequencies) were calculated for the variables. Confirmatory factor analyses (CFA) were conducted to assess the factor structure of the HEXACO-60 and the AMQ (23 misbehaviour items). The validation study of the AMQ has been submitted for publication elsewhere. Comparative fit index (CFI), root mean square error of approximation (RMSEA), chi-square test with degrees of freedom, χ²_(df)_, and standardised factor loadings were analysed as model fit indicators. Sum scores were computed for the HEXACO dimensions (after reverse coding negatively formulated items), the AMQ and its eight dimensions. Higher scores indicate higher levels of each personality dimension and academic misconduct, respectively. The internal consistency of the scales was assessed using Cronbach’s alpha (α).

Cluster analysis was conducted to categorise students into personality types according to their standardised scores (z*-*scores) in the HEXACO dimensions (*M* = 0 and *SD* = 1). These were preferred to raw scores to mitigate the influence of dimensions with larger standard deviations on cluster formation [34, 35]. Three hierarchical agglomerative clustering methods were initially examined to identify the possible number of cluster solutions, with Ward’s method being the most satisfactory. The cluster solutions identified in Ward’s dendrogram were then analysed through iterative *k*-means clustering to obtain the final cluster centres (mean z-scores of HEXACO dimensions). This method produces homogenous groups and is less affected by outliers than hierarchical methods [47]. The solution that provided the best differentiation between clusters, more interpretable groups, and sufficient stability was adopted.

After clustering the students based on HEXACO personality, further analyses were conducted to compare the clusters regarding their personality dimensions, as well as other variables that were not used in the clustering process. One-way analysis of variance (ANOVA) with post-hoc Tukey test was conducted to assess differences between the clusters in HEXACO dimensions (final cluster centres), self-reported academic misconduct and perceptions of peer fraud, severity of penalty and knowledge of the academic code. Bar chart illustrations of final cluster centres and self-reported academic misbehaviour were based on mean z-scores to facilitate interpretation. Pearson’s chi-square test of independence (χ²) was used to assess whether the personality type (cluster) of the students was independent of their sociodemographic and academic characteristics. The significance level was set at *p* < 0.05. CFA were performed in R software version 4.4.1. Other statistical analyses were conducted in SPSS Statistics Base version 29.0.

## Results

Of the 1,398 students who completed the questionnaire, some opted to omit responses for gender (*N* = 17), parental education (*N* = 14), and school funding concern (*N* = 36), being treated as missing values. Of the 1,381 students who indicated their gender, 71.7% were females, 27.5% were males, and 0.8% selected ‘other’. Regarding parental education, 16.4% reported elementary/lower, 29.6% secondary education, 31.6% bachelor’s degree, and 22.4% master’s/doctorate. Over half of the students expressed some/high concern (56.8%) about having sufficient funding to complete their degree. The majority had attended public secondary schools (75.5%). The students were enrolled in the first (48.1%), second (19.2%), and final or integrated master’s years (3rd to 6th year, 32.7%) of various fields of study: 31.4% in Health (and Sports) Sciences, 10.7% in Sciences (Natural/Exact), 13.4% in Engineering/Technology, 17.0% in Arts/Humanities (including Literature), 13.6% in Economics/Law (including Management and Criminology), and 11.2% in Social Sciences.

### Factor structure and reliability of the scales

The six-factor solution validated in the literature [45] for the HEXACO-60 was confirmed with similar results: CFI = 0.820, RMSEA = 0.041, and χ²_(1535)_ = 5197.97 (*p* < 0.001), after covarying error terms on items within the same factor based on modification indices. However, two items were removed: “I tend to be lenient in judging other people” (Agreeableness) and “I think that paying attention to radical ideas is a waste of time” (Openness), as they had factor loadings <|0.20| (loadings >|0.30| for the remaining items, all significant at *p* < 0.001). The scores for these two traits were computed using 9 items each, instead of the original 10. CFA using academic misconduct (AMQ) as a second-order factor comprising eight dimensions showed a good model fit with CFI = 0.972, RMSEA = 0.038, χ²_(225)_ = 672.04 (*p* < 0.001) and factor loadings ≥ 0.50 (*p* < 0.001). The CFA model is presented in the Electronic Supplementary Materials (ESM)– Fig. S1. The HEXACO dimensions (α = 0.72–0.83), the AMQ (α = 0.70) and its dimensions (α = 0.58–0.84, except for Impersonation, with α = 0.45) showed suitable internal consistency.

### Cluster analysis of the HEXACO personality dimensions

For consistency, the personality types will be referred to as personality clusters in the results section. Hypotheses 1 and 2 were tested using cluster analysis. The dendrogram produced by Ward’s hierarchical method (ESM– Fig. S2) suggested solutions of three to six clusters, which were examined through *k*-means clustering. The six-cluster solution was discarded due to its reduced stability and limited interpretability. The three-cluster solution produced two well-adjusted personality clusters and one maladjusted cluster, with below-average scores in all HEXACO dimensions. From solutions three to five, more distinctive clusters emerged, showing increasing stability. From solutions three to five, no cases from the maladjusted cluster in solution three were added to the well-adjusted clusters in solution five (comparisons between cluster solutions are presented in ESM– Table S1). Therefore, the five-cluster solution was adopted as it showed the best differentiation between clusters, yielded interpretable groups, and demonstrated sufficient stability.

**Fig. 1:**
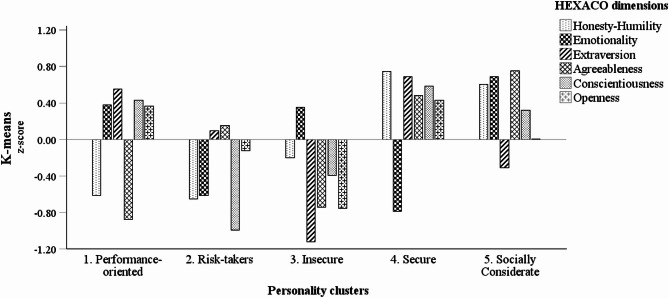
Mean z-scores of the HEXACO dimensions for the five personality clusters

The five clusters were characterised as follows: (1) Performance-oriented, showing well below-average Agreeableness and Honesty-Humility, and above-average scores in the other dimensions; (2) Risk-takers, displaying well below-average Conscientiousness (lowest among all clusters), Honesty-Humility (among the lowest), and Emotionality, and relatively average scores in the remaining dimensions; (3) Insecure, exhibiting above-average Emotionality and below-average levels in the other dimensions, particularly Extraversion (lowest among all clusters); (4) Secure, featuring well below-average Emotionality and above-average scores in all other dimensions, especially Honesty-Humility and Extraversion; and (5) Socially Considerate, presenting above-average Agreeableness, Emotionality (highest among all clusters), Honesty-Humility (among the highest), and Consciousness, average Openness, and below-average Extraversion. Personality cluster sizes ranged from 17.7 to 22.1% of the sample. Differences in the mean z-scores of the HEXACO dimensions (final cluster centres) between the five clusters were assessed using ANOVA (see ESM– Table S2) and are illustrated in Fig. 1.

### Sociodemographic and academic distribution of the personality clusters

Table 1 presents the distribution of the personality clusters across sociodemographic groups, academic years and fields of study, along with chi-square test (χ²) results. The observed variations across gender (*p* < 0.001), fields of study (*p* < 0.001), and school funding concern (*p* = 0.002) supported hypothesis 3. Most females were categorised as Socially Considerate (26.6%) and Performance-oriented (22.0%), followed by Insecure (18.8%). In turn, male students were predominantly classified as Risk-takers (36.3%) and Secure (27.6%).

The personality clusters showed a more even distribution in Health Sciences (Socially Considerate and Secure (21.4%) slightly overrepresented) than among non-health fields. The Socially Considerate cluster was the most prevalent in Sciences (27.5%) and Social Sciences (31.8%), while Risk-takers were overrepresented in Engineering/Technology (34.0%). Secure and Risk-takers (23.6% each) were the most prevalent clusters in Arts/Humanities, while Performance-oriented (24.7%) was the most represented among Economics/Law students. The interpretation of field of study differences should consider gender distribution inequalities across fields. Although female students were globally overrepresented, Engineering/Technology and Economics/Law exhibited the highest proportions of males, while Social Sciences had the lowest (ESM– Table S3).

Finally, among students who expressed school funding concerns, the Socially Considerate (24.0%) cluster was the most prevalent. Conversely, Risk-takers (24.0%) and Secure (22.3%) were the most represented among those who stated no funding concern, while Insecure was the lowest (14.5%). The personality cluster was not associated with academic year (*p* = 0.241), type of secondary school (*p* = 0.809), and level of parental education (*p* = 0.072), although the latter approached significance.

**Table 1:** Sociodemographic and academic distribution of the personality clusters

		Personality cluster (%)											
Variable (valid N)		1. Performance-oriented		2. Risk-takers		3. Insecure		4. Secure		5. Socially Considerate		Total (%)	χ²_(df)_
Gender													
Female (990)		22.0%		14.6%		18.8%		18.0%		26.6%		100.0%	χ²_(4)_ = **127.23**
Male (380)		12.4%		36.3%		13.9%		27.6%		9.7%		100.0%	
Field of study													
Health (477)		19.1%		18.9%		17.0%		21.4%		23.7%		100.0%	χ²_(20)_ = **67.82**
Sciences (149)		19.5%		14.8%		24.8%		13.4%		27.5%		100.0%	
Engineering/Technology (188)		11.2%		34.0%		16.5%		21.3%		17.0%		100.0%	
Arts/Humanities (237)		19.4%		23.6%		15.2%		23.6%		15.8%		100.0%	
Economics/Law (190)		24.7%		20.0%		15.8%		23.7%		15.8%		100.0%	
Social Sciences (157)		21.0%		11.5%		20.4%		15.3%		31.8%		100.0%	
School funding concern													
None (588)		20.2%		24.0%		14.5%		22.3%		19.0%		100.0%	χ²_(4)_ = **16.43**
Some/High (774)		18.9%		17.8%		19.1%		19.6%		24.5%		100.0%	
Academic year													
1st year (672)		16.7%		21.1%		18.5%		19.9%		23.8%		100.0%	χ²_(8)_ = 10.35
2nd year (269)		18.6%		20.8%		19.3%		19.7%		21.6%		100.0%	
≥ 3rd year (457)		23.0%		19.7%		15.5%		21.9%		19.9%		100.0%	
Secondary school													
Public (1,055)		19.0%		20.9%		17.4%		20.0%		22.7%		100.0%	χ²_(4)_ = 1.60
Private (343)		19.5%		19.5%		18.4%		22.2%		20.4%		100.0%	
Parental education level													
Elementary/lower (227)		18.5%		14.5%		17.6%		21.6%		27.8%		100.0%	χ²_(12)_ = 19.75
Secondary education (410)		19.0%		23.9%		16.3%		21.0%		19.8%		100.0%	
Bachelor’s degree (437)		18.8%		18.5%		20.1%		18.5%		24.0%		100.0%	
Master’s/Doctorate (310)		21.0%		22.9%		15.5%		22.3%		18.4%		100.0%	

Note: Pearson’s chi-square test (χ²) results with significant values (all at *p* < 0.01) are highlighted in bold

### Personality cluster differences in academic misconduct

Differences between personality clusters in mean scores of self-reported academic misconduct– AMQ and its eight dimensions– and in perceptions of peer fraud, severity of penalty, and knowledge of the academic code were assessed using ANOVA (ESM– Table S4). On the overall measure of misconduct (AMQ sum score), Risk-takers disclosed the highest levels (*p* < 0.001), with Performance-oriented (*p* < 0.001) and Insecure (*p* < 0.05) scoring above Secure and Socially Considerate clusters. These differences are illustrated in ESM– Fig. S3.

Regarding the forms of misconduct, Risk-takers disclosed the highest involvement in Plagiarism, Fraud in Academic Work, Not Reporting peer misconduct (*p* < 0.01) and Impersonation (*p* < 0.05). Risk-takers scored higher than Insecure, Secure and Socially Considerate in both forms of Exam Cheating (using notes/device and with colleagues) (*p* < 0.001). Risk-takers also scored higher than Secure and Socially Considerate in the remaining forms of misconduct (*p* ≤ 0.01), except Obtaining/providing information prior exams, where the difference to Socially Considerate was just above 0.05.

Performance-oriented reported greater engagement in Exam Cheating (both forms) and Signature Forgery than Secure (*p* < 0.05) and Socially Considerate (*p* < 0.01). They scored higher than Secure in Plagiarism (*p* < 0.001) and Obtaining/providing information prior exams (*p* < 0.05), and higher than Socially Considerate in Not Reporting peer misconduct (*p* ≤ 0.01).

**Fig. 2:**
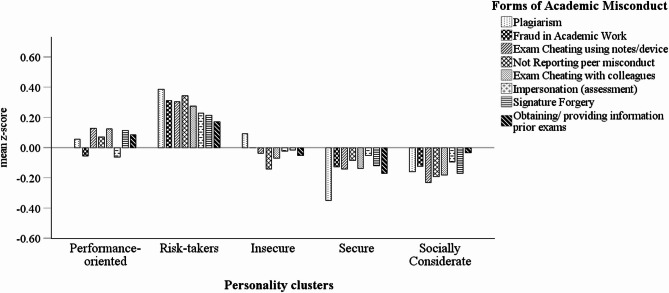
Mean z-score differences in self-reported engagement in the eight forms of academic misconduct across personality clusters

Insecure students disclosed greater Plagiarism than Secure (*p* < 0.001) and Socially Considerate (*p* < 0.05), while no differences were observed in the remaining misbehaviours (*p* > 0.05). The differences in scores for the eight forms of academic misconduct across personality clusters are illustrated in Fig. 2.

Regarding student perceptions, Performance-oriented perceived the highest levels of peer fraud (*p* < 0.02). Risk-takers perceived lower penalties for cheating compared to Insecure, Socially Considerate (*p* < 0.001) and Performance-oriented (*p* < 0.02), with the difference to Secure being just above 0.05. Risk-takers and Insecure disclosed lower knowledge of the academic code compared to Secure (*p* < 0.01). The difference between Insecure and Socially Considerate was just above 0.05, with the latter stating slightly greater knowledge of the code. Overall, the results reported above supported hypothesis 4.

## Discussion

This study identified five distinct personality types, described as Performance-Oriented, Risk-Takers, Insecure, Secure and Socially Considerate, which differed in self-reported academic misconduct and related perceptions. The prevalence of the personality types also varied across gender, school funding concern, and field of study.

### Five personality types based on the HEXACO traits

The identified number of personality types aligned with previous studies [37, 38]. Additionally, the types largely resembled others in the literature, namely the Self-Confident, Goal-oriented, Socially Considerate [38], Insecure, and Achievement-oriented agentic [37] groups. These findings corroborated hypotheses 1 and 2. However, while previous research reported unbalanced personality groups (6–40% of the sample), this study yielded relatively uniform-sized groups (around 20% each). This discrepancy is potentially attributable to differing statistical methodologies (latent profile analysis vs. cluster analysis). Studies that analysed personality types by combining six-factor dimensions with other personality models [43, 48] provided limited comparability.

### Prevalence of the personality types across sociodemographic groups and fields of study

Consistent with the literature [37, 39], personality types that displayed anxiety, risk aversion and prosocial dispositions were more prevalent among females, while Risk-takers and Secure were more common among male students. As anticipated [40, 41, 49], personality type distribution variated across fields of study. While the five types were relatively evenly represented in Health Sciences, a more heterogeneous distribution was observed among non-health fields. This could be explained by the overrepresentation of Health students (around 30% of the sample), since the method of clustering analysis produced relatively uniform-sized groups. Similar to Health Sciences, the prosocial type was the most prevalent in Sciences and Social Sciences, corroborating prior literature [49]. In Engineering/Technology, which had the highest proportion of males, Risk-takers were markedly the most common. The two types showing the lowest levels of Emotionality (Risk-takers and Secure) were the most prevalent in Arts/Humanities. Although it contrasts with prior findings [40, 49], there is evidence suggesting a reduced propensity of these students to experience anxiety [41]. In turn, the performance-focused type was the most prevalent in Economics/Law, a field linked to a higher prevalence of competitive and individualistic traits [41, 49]. Personality was not associated with academic year, potentially due to the reduced age range [15]. School funding concern was the only socio-economic indicator that showed personality type variations, possibly being the strongest indicator of economic status. Conversely to well-adjusted and risk-seekers, prosocial and anxiety-prone types seemed more associated with less favourable economic backgrounds. The above-described findings supported hypothesis 3.

### Personality type differences in academic misconduct and related perceptions

Confirming the final hypothesis, self-reported misconduct varied between personality types. Risk-takers disclosed the highest levels of misconduct, particularly in severe forms. They shared traits associated with risk-taking and impulsivity [19], which have been linked to academic misbehaviour [30, 31, 50]. The lack of prudence, responsibility, self- and moral restraint that seem to characterise this personality type can contribute to academic procrastination [29] and impulsive misbehaviour [15, 30]. This is potentially heightened by a sense of impunity, as reflected by their perceptions of low penalties for cheating. Risk-takers can benefit from external structuring strategies, such as planning lists, intermediate goals and regular deadlines, which can enhance prioritisation, organisation and time-management skills, reducing procrastination [28, 29].

Performance-oriented scored lower than Risk-takers in overall misconduct and half of its forms, potentially due to their higher Conscientiousness. Conversely, they reported globally greater misconduct than Secure and Socially Considerate. Performance-oriented characteristics may predispose an increased focus [19], and possibly anxiety [38], concerning individual achievement. Their lower levels in prosocial traits may also lead to a reduced sensitivity to the social injustice of cheating. Additionally, consistent with prior research [51], this type perceived the highest levels of peer fraud, suggesting that they may view their academic setting as more tolerant of cheating [52]. Performance-oriented students can benefit from strategies that foster intrinsic learning motivation, reduce performance anxiety, and emphasise the benefits of collaborative learning [28]. These strategies may include clearly articulating the relevance of learning objectives for future practice, encouraging idea sharing and facilitating group activities beyond evaluation [53].

Insecure students combined traits associated with psychological distress, anxiety, and decreased task orientation [19], which may hinder their ability to complete academic tasks [37]. They were among the lowest scorers in most forms of misconduct, except Plagiarism. Despite their potential aversion to the risks inherent in cheating, they may engage in certain misbehaviours due to a lack of confidence in their skills [19], performance difficulties [2, 32] or fear of failure [28]. These challenges may be further exacerbated by academic pressures, given the Insecure type was more prevalent among students with economic concerns. Therefore, interventions can focus on enhancing psychological well-being and resilience, academic (e.g., academic writing) and decision-making skills [28], and self-confidence in academic task completion [54].

Finally, Socially Considerate and Secure students disclosed the lowest misconduct rates. Secure exhibited the most well-adjusted personality type [37, 38] and seemed linked to a slightly more positive social context, being the second most prevalent among students without financial concerns. Secure also reported greater knowledge of the code than some of the others, possibly reflecting self-confidence in their skills. Notably, in prior research [29], the closest FFM type (Resilients) did not differ from Undercontrollers (the closest to Risk-takers) in self-reported cheating. Finally, the cooperative, prosocial and risk-averse characteristics inherent in the Socially Considerate type may suggest a stronger sense of justice. Given the unethical and unfair implications of cheating, this may help to explain that, in line with prior studies [43], this type was one of the less likely to disclose misbehaviour.

### Limitations and future directions

This cross-sectional study was conducted at one national university, even though fourteen faculties were represented and participants attended different majors and academic years. This diversity reduced the risk of homogeneity of personality traits in the sample. In turn, imbalances, for example, in gender representation across fields of study, should be considered when interpreting the results. Potential social desirability bias associated with self-report questionnaires was addressed by guaranteeing anonymity and confidentiality [55] and encouraging participants to respond according to their actual behaviours and feelings [56]. The questionnaire also asked about past misbehaviours instead of specific or recent incidents to reduce the fear of repercussions. Additionally, personality may influence the tendency to self-report misconduct [43]. At this level, personality associations with both self-reported and observed misbehaviour revealed largely similar patterns [43, 55].

Although the clustering method is data-driven and can be subject to sample characteristics, the five types obtained largely replicate the literature. Finally, few studies have associated personality types with misconduct in university students and their types derived from distinct trait combinations– FFM [29] and HEXACO with Dark Triad [43]. Therefore, the personality types here analysed provided novel information. This study also advances prior research by assessing different forms of misconduct and related perceptions. Furthermore, analysing type distribution across sociodemographic groups and fields of study offered relevant insights into ‘who’ and ‘where’ they were. Future research should focus on exploring how different psychosocial and academic characteristics of the students interact to predict misconduct. This data should contribute to proposing more targeted interventions to support students with different personality types to avoid misconduct.

## Conclusions

This study builds on previous research by analysing a distinct set of personality types in association with various forms of academic misconduct. The five types displayed interesting psychosocial differences that shed light on their potential academic needs. The findings and recommendations presented in this study can facilitate knowledge transfer aimed at designing pedagogical approaches and support systems that help different personality types in health and non-health fields comply with academic integrity.

## Electronic supplementary material

Below is the link to the electronic supplementary material.

Supplementary Material 1

## Data Availability

Data for this study can be made available on request to the authors.

## Abbreviations

ANOVA: Analysis of Variance
AMQ: Academic Misconduct Questionnaire
CFA: Confirmatory Factor Analysis
CFI: Comparative Fit Index
DF: Degrees of Freedom
RMSEA: Root Mean Square Error of Approximation
SD: Standard Deviation
Z-scores: Standardised Scores
