# Supplementary material for: Identifying HEXACO personality types: what do type characteristics tell us about student misconduct?
Source: BMC Med Educ. 2025 Jul 19;25:1083. doi: 10.1186/s12909-025-07599-5 (PMC12275385; doi:10.1186/s12909-025-07599-5)
Supplement: Supplementary file 1 — Supplementary Material 1 [file 12909_2025_7599_MOESM1_ESM.docx]

**Electronic Supplementary Material (ESM)**


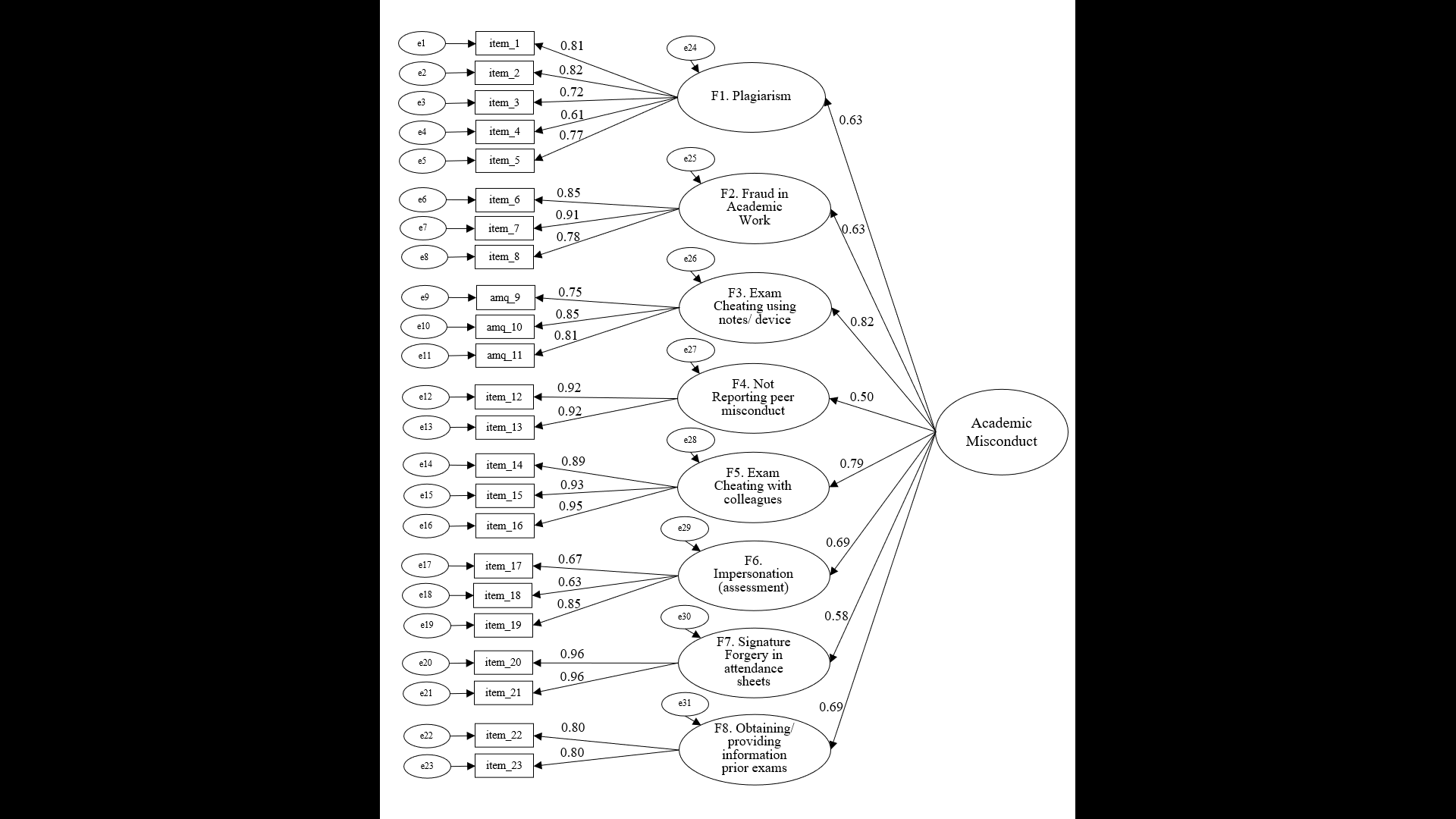


**Fig. S1** Confirmatory factor analysis model of the Academic Misconduct Questionnaire (AMQ)


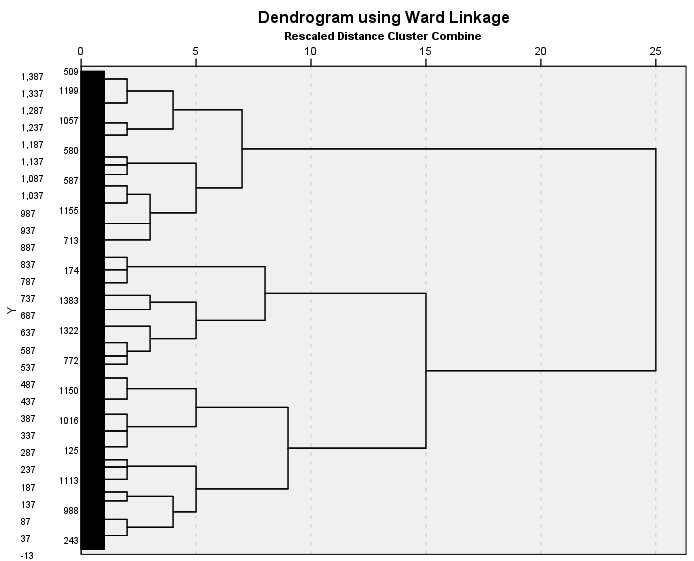


**Fig. S2** Dendrogram illustrating hierarchical clustering using Ward’s method


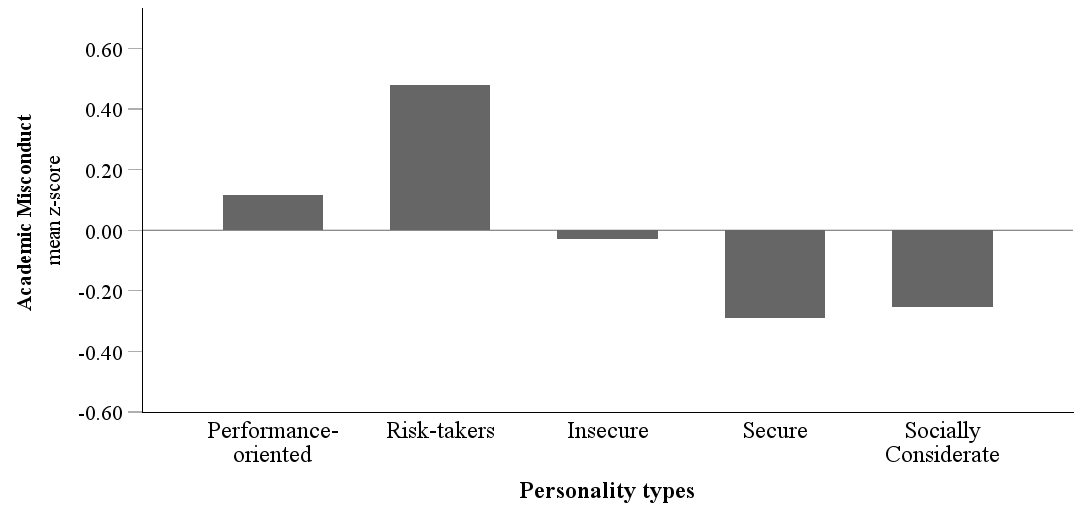
**Fig. S3** Mean z-score differences in self-reported engagement in academic misconduct (AMQ sum score) across personality clusters

**Table S1** Comparison of case changes between cluster solutions

| Three- to four-cluster solutions | | | | | | | | |
| --- | --- | --- | --- | --- | --- | --- | --- | --- |
|  | **1^b^** | **2^a^** | | **3^d^** | **4^c^** | |  |  |
| **1^a^** | 3.2% | 76.0% | | 56.9% | 0.0% | |  |  |
| **2^b^** | 93.6% | 0.0% | | 26.6% | 13.3% | |  |  |
| **3^c^** | 3.2% | 24.0% | | 16.6% | 86.7% | |  |  |
| Total | 100% | 100% | | 100% | 100% | |  |  |
| Four- to five-cluster solutions | | | | | | | | |
|  | **1^d^** | **2^e^** | | **3^a^** | **4^b^** | | **5^c^** |  |
| **1^b^** | 6.4% | 26.7% | | 0.0% | 92.0% | | 6.1% |  |
| **2^a^** | 1.9% | 53.1% | | 81.0% | 0.0% | | 1.6% |  |
| **3^d^** | 82.0% | 19.8% | | 4.9% | 0.0% | | 0.6% |  |
| **4^c^** | 9.7% | 0.3% | | 14.2% | 8.0% | | 91.6% |  |
| Total | 100% | 100% | | 100% | 100% | | 100% |  |
| Three- to five-cluster solutions | | | | | | | | |
|  | **1^d^** | **2^e^** | | **3^a^** | **4^b^** | | **5^c^** |  |
| **1^a^** | 42.3% | 68.4% | | 57.9% | 0.0% | | 0.0% |  |
| **2^b^** | 32.6% | 25.7% | | 0.0% | 97.2% | | 12.6% |  |
| **3^c^** | 25.1% | 5.9% | | 42.1% | 2.8% | | 87.4% |  |
| Total | 100% | 100% | | 100% | 100% | | 100% |  |
| Five- to six-cluster solutions | | | | | | | | |
|  | **1^d^** | **2^c^** | **3^a^** | | **4^e^** | **5^b^** | | **6^f^** |
| **1^d^** | 73.5% | 2.4% | 1.3% | | 32.3% | 0.5% | | 4.1% |
| **2^e^** | 0.4% | 0.4% | 8.2% | | 64.2% | 19.4% | | 26.1% |
| **3^a^** | 2.7% | 0.0% | 85.3% | | 2.7% | 0.5% | | 14.9% |
| **4^b^** | 17.9% | 22.4% | 0.0% | | 0.4% | 76.6% | | 15.4% |
| **5^c^** | 5.4% | 74.7% | 5.2% | | 0.4% | 3.0% | | 39.4% |
| Total | 100% | 100% | 100% | | 100% | 100% | | 100% |

*Note:* For each pair of cluster solutions compared, exponentials ^a,b,c,d,e,f^ indicate the corresponding groups between clusters (e.g., Cluster 1 in the four-cluster solution derived from Cluster 2 in the three-cluster solution; Cluster 3 in the four-cluster solution is a newly emerged cluster compared to the solution of three).

Five-cluster solution (adopted in this study): 1- Performance-oriented, 2- Risk-takers, 3- Insecure, 4- Secure and 5- Socially Considerate.

**Table S2** Analysis of Variance (ANOVA) examining differences in mean z-scores of HEXACO dimensions (final cluster centres) across personality clusters

|  |  | Personality clusters (%) | | | | | | |  |
| --- | --- | --- | --- | --- | --- | --- | --- | --- | --- |
| HEXACO dimension,  Mean (SD) | | 1  Performance-oriented | 2  Risk-takers | | 3  Insecure | 4  Secure | | 5  Socially Considerate | ANOVA  (*F*)^†^ |
|  |  | (19.1%) | (20.6%) | | (17.7%) | (20.5%) | | (22.1%) |  |
| Honesty-Humility,  35.59 (6.33) | | -0.61 ^a^ | -0.65 ^a^ | -0.20 | | | 0.75 ^b^ | 0.60 ^b^ | 197.51 |
| Emotionality,  33.89 (6.56) | | 0.38 ^a^ | -0.61 ^b^ | 0.35 ^a^ | | | -0.79 ^b^ | 0.69 | 196.02 |
| Extraversion,  31.23 (7.05) | | 0.55 ^a^ | 0.09 | -1.12 | | | 0.69 ^a^ | -0.31 | 232.01 |
| Conscientiousness, 36.84 (5.88) | | 0.43 ^a,b^ | -0.99 | -0.39 | | | 0.59 ^a^ | 0.32 ^b^ | 195.71 |
| Agreeableness,  27.87 (5.32) | | -0.87 ^a^ | 0.15 | -0.74 ^a^ | | | 0.48 | 0.75 | 254.78 |
| Openness,  32.59 (5.99) | | 0.36 ^a^ | -0.12 ^b^ | -0.76 | | | 0.43 ^a^ | 0.01 ^b^ | 70.11 |

^†^ All values are significant at *p* < 0.001.

*Note:* In each row, cells with exponentials ^a,b^ indicate mean z-scores that were not significantly different (*p* > 0.05) between clusters in ANOVA post-hoc Tukey comparisons (e.g., there was no significant difference in mean z-scores of Honesty-Humility between clusters 1 and 2, and between clusters 4 and 5). Cell values without exponentials were significantly different (*p* < 0.05) from all other values in the same row.

**Table S3** Pearson's chi-square test of independence between gender and field of study

|  | Gender | | χ²_(df)_  *p*-value |
| --- | --- | --- | --- |
| Field of study | Female | Male |  |
| Health Sciences | 75.3% | 24.7% | χ²_(5)_ = 40.45  < 0.001 |
| Sciences | 71.7% | 28.3% |  |
| Engineering/ Technology | 59.0% | 41.0% |  |
| Arts/ Humanities | 72.8% | 27.2% |  |
| Economics/ Law | 65.1% | 34.9% |  |
| Social Sciences | 87.4% | 12.6% |  |

**Table S4** Analysis of Variance (ANOVA) examining differences in academic misconduct and related perceptions across personality clusters

| Variable, *Mean* (*SD*) | 1.  Performance-  oriented | 2.  Risk-Takers | 3.  Insecure | 4.  Secure | 5.  Socially Considerate | ANOVA  (*F*)^†^ |  |
| --- | --- | --- | --- | --- | --- | --- | --- |
| Academic Misconduct (AMQ), 30.08 (4.45) | 30.61^a^ | 32.22 | 29.95 ^a^ | 28.79 ^b^ | 28.95 ^b^ | 31.12 |  |
| Plagiarism, 7.41 (1.68) | 7.50 ^a, b^ | 8.06 | 7.56 ^a^ | 6.82 ^c^ | 7.14 ^b, c^ | 23.83 |  |
| Fraud in Academic Work, 3.18 (0.53) | 3.15 ^a^ | 3.35 | 3.18 ^a^ | 3.11 ^a^ | 3.12 ^a^ | 9.80 |  |
| Exam Cheating using notes/device,  3.60 (0.89) | 3.72 ^a, b^ | 3.87 ^a^ | 3.57 ^b, c^ | 3.47 ^c^ | 3.39 ^c^ | 14.02 |  |
| Not Reporting peer misconduct, 2.40 (0.72) | 2.45 ^a^ | 2.65 | 2.30 ^a, b^ | 2.34 ^a, b^ | 2.27 ^b^ | 13.94 |  |
| Exam Cheating with colleagues, 4.53 (1.24) | 4.69 ^a, b^ | 4.87 ^a, b^ | 4.45 ^b, c^ | 4.36 ^c^ | 4.31 ^c^ | 11.03 |  |
| Impersonation (assessment), 3.25 (0.56) | 3.22 ^a^ | 3.38 | 3.24 ^a^ | 3.22 ^a^ | 3.20 ^a^ | 5.03 |  |
| Signature Forgery in attendance sheets,  2.76 (0.90) | 2.87 ^a^ | 2.95 ^a^ | 2.75 ^a, b^ | 2.66 ^b^ | 2.61 ^b^ | 7.59 |  |
| Obtaining/ providing information prior exams, 2.95 (0.82) | 3.01 ^a^ | 3.09 ^a^ | 2.90 ^a, b^ | 2.80 ^b^ | 2.92 ^a, b^ | 4.98 |  |
| Peer fraud, 2.84 (0.97) | | 3.07 | 2.79 ^a^ | 2.79 ^a^ | 2.81 ^a^ | 2.77 ^a^ | 4.43 |
| Severity of penalty,  3.63 (0.92) | | 3.66 ^a^ | 3.42 ^b^ | 3.74 ^a^ | 3.62 ^a,b^ | 3.73 ^a^ | 5.82 |
| Knowledge of academic code, 2.80 (0.87) | | 2.81 ^a,b^ | 2.71 ^a^ | 2.66 ^a^ | 2.96 ^b^ | 2.85 ^a,b^ | 5.14 |

^†^ All values are significant at *p* ≤ 0.001.

*Note:* In each row, cells with exponentials ^a,b,c^ indicate means that were not significantly different (*p* > 0.05) between clusters in ANOVA post-hoc Tukey comparisons (e.g., there was no significant difference in mean scores of AMQ between clusters 1 and 3, and between clusters 4 and 5). Cell values without exponentials were significantly different (*p* < 0.05) from all other values in the same row.
